# Statistical analysis plan for the randomized controlled trial CardioCare MV investigating a novel integrated care concept (NICC) for patients suffering from chronic cardiovascular disease

**DOI:** 10.1186/s13063-020-4052-6

**Published:** 2020-02-03

**Authors:** Andreas Ziegler, Miriam Mann, Bernard Brandewiede, Hermann Dittrich, Sissy Hintz, Katja Krockenberger, Alper Öner, Marcos Oliviera de Sousa, Christian Schmidt

**Affiliations:** 1 StatSol, Moenring 2, 23560 Lübeck, Germany; 2 School of Mathematics, Statistics and Computer Science, University of KwaZulu-Natal, Pietermaritzburg, South Africa; 3 Present Address: Cardio-Care AG, Medizincampus Davos, Herman Burchard Str. 1, 7265 Davos, Switzerland; 4 Universitätsmedizin Rostock Versorgungsstrukturen GmbH, Ernst-Heydemann-Str. 8, 18057 Rostock, Germany; 5 AMEDON GmbH, Willy-Brand-Allee 31c, 23554, Lübeck, Germany; 6 Abteilung Kardiologie, Universitätsmedizin Rostock, Ernst-Heydemann-Str. 8, 18057 Rostock, Germany; 7 Universitätsmedizin Rostock, Ernst-Heydemann-Str. 8, 18057 Rostock, Germany

**Keywords:** Atrial fibrillation, Care center, Disease management program, Evidence-based care, Heart failure, Hospitalization, Integrated care, Randomized controlled trial, Telemedicine, Treatment-resistant hypertension

## Abstract

**Background:**

Cardiovascular diseases are the major cause of death globally and represent a major economic burden on health care systems. For patients with heart failure, atrial fibrillation or therapy-resistant hypertension, we have developed a novel integrated care concept (NICC) which combines telemedicine with intensive support by a care center, including a call center, an integrated care network including inpatient and outpatient care providers and guideline therapy for patients (Schmidt et al. 2018 Trials 19:120). Here, we describe challenges and solutions in patient recruitment and provide the statistical analysis plan.

**Methods:**

The study CardioCare MV is a prospective, randomized, controlled, parallel-group, open-label, bi-center trial with two groups for comparing NICC with standard of care (SoC). Because of issues with patient enrollment we adapted the study plan after consultation with the Ethics Committee and the funding agency. We altered the analysis strategy for the primary endpoints, which led to a change in the required sample size. We also changed the access points to patients from inpatient hospitals specialized in the treatment of patients with cardiovascular disease to specialized practices.

**Results:**

Recruitment of patients started on 1 December 2017, and first patient in was on 4 December 2017. Recruitment was completed on 15 August 2019 as planned according to the amended study plan. The follow-up period will end in August 2020. A total of 964 patients was enrolled into the trial. The statistical analysis plan was finalized prior to last patient in. Results will be available by the end of 2020.

**Discussion:**

The trial will inform care providers whether quality of care can be improved by an integrated care concept providing telemedicine through a round-the-clock call center approach. We expect that cost of the NICC will be lower than standard care because of reduced hospitalizations. The trial will guide additional research to disentangle the effects of this complex intervention.

**Trial registration:**

DRKS, ID: DRKS00013124. Registered on 5 October 2017

ClinicalTrials.gov, ID: NCT03317951. Registered on 17 October 2017

## Main text

The study protocol of the randomized controlled trial CardioCare MV was published in 2018 in this journal [1]. The aim of the trial is to show that a novel integrated care concept (NICC) is superior to standard of care (SoC) in patients with atrial fibrillation (AF), heart failure (HF) or treatment-resistant hypertension (TRH). NICC is a combination of telemedicine with intensive support by a care center, including a call center, an integrated care network including inpatient and outpatient care providers and guideline therapy for patients. This combination of interventions may increase compliance and may allow for early interventions before a serious event has occurred.

Recruitment started on 1 December 2017, and the aim was the inclusion of a total of approximately 2930 patients, i.e., approximately 1465 patients per treatment group within a 15-month timeframe [1]. Patients should have been primarily recruited from two hospitals in Mecklenburg-Western Pomerania, namely the Clinic of Cardiology at the University Medical Center Rostock (UMR) and the Cardiology Department at the Helios Clinic in Schwerin. However, after a few months of recruitment and projection of recruitment we concluded that recruitment was too slow to achieve the targeted sample size within the intended 15-month period. In the next section, we sketch reasons for the delay and summarize our lessons learned from this unfortunate recruitment experience.

In order to avoid early termination of the trial, the sponsor, the project management and the trial statistician initiated discussions with the parties involved the study: the Joint Federal Committee, which is the funder of the project, the Data Monitoring Committee (DMC), the Ethics Committee of the Medical Faculty of the University of Rostock and the participating health insurances whether a sample size reduction would be feasible in order to complete the trial successfully within the funding period, which ends by the end of 2020.

In these discussions, three key issues were identified:

1. Prolongation of the recruitment period until 15 August 2019
2. Inclusion of 26 cardiologists and internists specialized in cardiology from the state of Mecklenburg-Western Pomerania as a part of the study center UMR
3. Change of the multiple testing procedure for the primary endpoints and replanning of the required sample size of the trial. This change in the multiple testing procedure will be described below

The new plan was presented to the DMC and the funder. Both parties agreed to the proposal (DMC on 6 June 2019, funder on 12 July 2019), which subsequently was submitted to the Ethics Committee of the Medical Faculty of the University of Rostock (amendment No. 2 to the study protocol, current version 3.0 of the study protocol). The Ethics Committee had no objections against the change of the plan (letter from the Ethics Committee as of 20 August 2019). In parallel to these changes of the study plan, the trial statistician (AZ) finalized the statistical analysis plan (SAP) on 7 August 2019. The SAP will be presented in the next-but-one section.

## Recruitment experience and lessons learned

CardioCare MV was originally planned as a general-practitioner-centered study with patient recruitment from hospitals. Specifically, inpatients treated in one of the two hospitals should have been invited to participate in the trial, and the assignment to one of the study centers should have happened through a direct link with the general practitioner. This approach failed because inpatients were too focused on their acute illness and corresponding hospital stay. Some patients even replied that they felt too ill for a participation in the trial. The reason is that a special sensibility and comprehensive educational effort is required to familiarize patients with this special form of care. We originally assumed that we would be able to enroll approximately every third inpatient meeting the inclusion criteria into the trial. Because of the specific funding of the trial and the project structure, we were only able to enroll members of the health insurance companies Allgemeine Ortskrankenkasse (AOK) Nordost and Techniker Krankenkasse (TK), which cover approximately half of all patients covered under the statutory insurance plan. As a result of these two restrictions, we could only recruit every 20th to 25th inpatient into the trial.

To increase recruitment, we conducted intensive public education campaigns, such as information booths and lectures at public events, had attendance calls in magazines, used information letters from health insurances, distributed information flyers in surgeries, hospitals, pharmacies and medical supply stores. This has led to an extension of the awareness level. Unfortunately, this effort did not lead to a substantial increase in the enrollment of patients into the trial.

In contrast, the visit of preventative heart sport groups in Mecklenburg-Western Pomerania led to a significant improvement in recruitment. Participants of these sport groups intensively deal with their illness, and they were very open to new care concepts.

The low outcome of our activities overall led to a reorganization of the recruitment activities. Specifically, the inclusion of established cardiologists and internists specialized in cardiology as part of the study center UMR led to an improved acceptance of the trial. One of the reasons for the higher acceptance was that patients were seriously ill inpatients. Another important reason is that patients do not have to travel to one of the two study center for their medical assessments, but standardized assessments are possible in the cardiology offices. With this action, the practicability of the project improved substantially, and, as a result, number of participants has been increased significantly within a few weeks. The revised required sample size (890 patients, see below) could be reached in the adapted recruitment period.

## Statistical analysis plan

### Hypotheses and original analysis strategy

In the study protocol, we described three primary endpoints for the trial:

1. First primary endpoint: composite endpoint
  The first primary endpoint is the composite endpoint of mortality, stroke and myocardial infarction, measured at 12 months after randomization
2. Second primary endpoint: hospitalization
  The second primary endpoint is the number of days spent in hospital, measured at 12 months after randomization as reported by patients
3. Third primary endpoint: composite endpoint
  The third primary endpoint is the composite endpoint of mortality, stroke, myocardial infarction and cardiac decompensation, measured at 12 months after randomization

Subsequently, three hypotheses were formulated for these three primary endpoints:

The hypothesis for the first primary endpoint is:

$$ {H}_0^1:{p}_A={p}_B\kern0.5em vs.\kern0.5em {H}_1^1:{p}_A\ne {p}_B, $$

where *p*_*A*_ is the event rate for the first primary endpoint in the NICC group, and *p*_*B*_ is the corresponding event rate in the SoC group 1 year after randomization.

The hypothesis for the second primary endpoint is:

$$ {H}_0^2:{\lambda}_A={\lambda}_B\kern0.5em vs.\kern0.5em {H}_1^2:{\lambda}_A\ne {\lambda}_B, $$

where *λ*_*A*_ is the mean number of hospitalizations in the NICC group, and *λ*_*B*_ is the mean number of hospitalizations in the SoC group within the 1-year observation period after randomization.

The hypothesis for the third primary endpoint is:

$$ {H}_0^3:{q}_A={q}_B\kern0.5em vs.\kern0.5em {H}_1^3:{q}_A\ne {q}_B, $$

where *q*_*A*_ is the event rate for the third primary endpoint in the NICC group, and *q*_*B*_ is the corresponding event rate in the SoC group 1 year after randomization.

The familywise error rate is set to 5%. All tests will be done two-sided. Originally, the following multiple testing procedure was aimed to be used [1]:

- Hypotheses *H*^1^ and *H*^2^ to be tested at the 2.5% test-level
- If *H*^1^ were significant at the two-sided 2.5% test-level, the full significance level of 2.5% would have fallen back to *H*^2^
- If *H*^2^ were significant at the two-sided 2.5% test-level, the full significance level of 2.5% would have fallen back to *H*^1^
- Only if both *H*^1^ and *H*^2^ were significant, *H*^3^ would have been tested at the 5% test-level

No other adjustments were planned for multiple testing. Neither interim analyses nor adaptations were planned for this trial.

### Revised analysis strategy

Since it turned out during recruitment that the originally planned number of patients cannot be reached within the funding period, the original analysis strategy was revised. This change in the sample size goes along with a change in the planned primary analysis. The familywise error rate is set to 5%, and all tests will be two-sided.

The following hierarchical multiple testing strategy will be used for the three primary endpoints instead of the original strategy:

- Step 1:
  - Case 1: *H*^2^ is significant at the two-sided 5% test-level. Then, the full significance level of 5% will fall to *H*^1^
  - Case 2: *H*^2^ is not significant at the two-sided 5% test-level. Then, the testing procedure will *stop*. None of the primary endpoints has demonstrated significance, and superiority of NICC over SoC has not been shown. One has to assume that NICC has the same hospitalization rate as SoC.
- Step 2:
  - Case 1: *H*^1^ is significant at the two-sided 5% test-level. Then, the full significance level of 5% will fall to *H*^3^
  - Case 2: *H*^1^ is not significant at the two-sided 5% test-level. Then, the testing procedure will *stop*. In this case, a difference in 1-year hospitalization rates between NICC and SoC has been demonstrated. However, the study failed to demonstrate superiority of NICC over SoC for the combined endpoint mortality, stroke and myocardial infarction. We have to assume that the rates in this combined endpoint are equal between the two groups
- Step 3:
  - Case 1: *H*^3^ is significant at the two-sided 5% test-level. Then, the study has demonstrated a difference in the hospitalization rates, the combined endpoint consisting in mortality, stroke and myocardial infarction and the combined endpoint consisting in mortality, stroke, myocardial infarction and cardiac decompensation between NICC and SoC at the 5% test-level
  - Case 2: *H*^3^ is not significant at the two-sided 5% test-level. Then, the study has demonstrated the difference in hospitalization rates and the difference in the rates for the combined endpoint between mortality, stroke and myocardial infarction between NICC and SoC at the 5% test-level. One has to assume that the event rates for the combined endpoint mortality, stroke, myocardial infarction and cardiac decompensation are identical between NICC and SoC

The confidence level for all hypotheses will be two-sided 95% because of the hierarchy of the tests.

Neither interim analyses nor adaptations are planned for this trial. As a result, there are no statistical rules for stopping the trial early. However, the sponsor has the right to terminate the trial prematurely if there are any relevant medical or ethical concerns, or if completing the trial is no longer practicable.

### Revised sample size and power calculation

This change in the analysis strategy goes along with a change in the sample size calculations. The assumptions made for these calculations are

- Allocation ratio 1:1
- Type I error level 0.05 two-sided
- Rate of hospitalizations per year in the NICC group: *λ*_*A*_ = 0.2
- Rate of hospitalizations per year in the SoC group: *λ*_*B*_ = 0.3
- Drop-out 8.5%

Then, the required total number of patients is approximately 890, i.e., 445 patients per group at 81.8% power. This total sample size leads to 80% power even if the drop-out is as large as 12.5%.

The R code for the power calculation is:

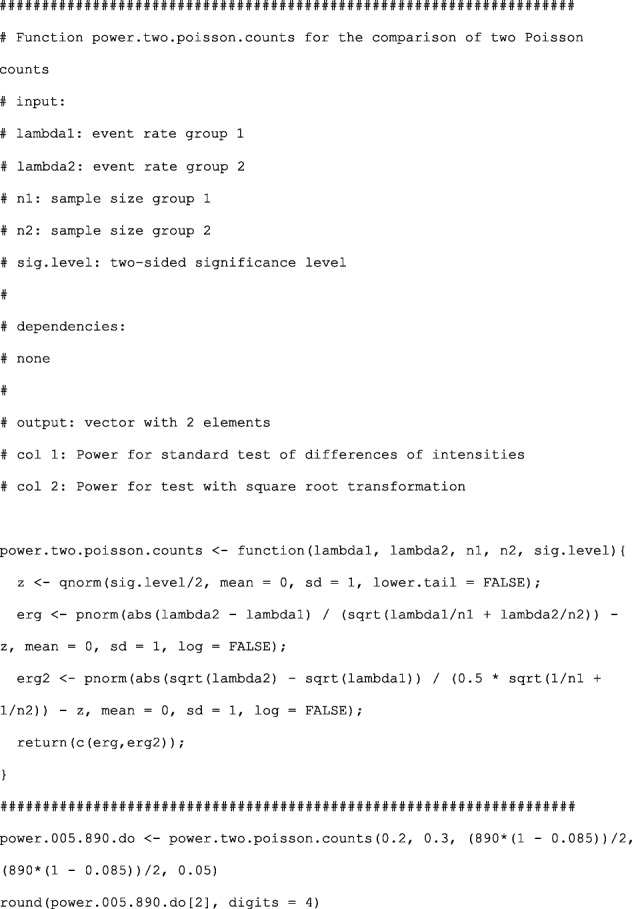



### Timing of outcome assessments and of final analysis

The schedule of assessments is described in Table 2 of [1]. The acceptable time window for data acquisition at both follow-up time points is ± 1 month. The final analysis will be performed after database closure.

### Analysis sets

Analysis populations for the primary endpoints will be the full analysis set (FAS) based on the intention-to-treat (ITT) principle. It consists of all patients receiving at least one treatment. For the NICC group, patients are included in the FAS dataset if the medical devices have been delivered to the patient. For the SoC group, patients are included in the FAS dataset after successful completion of the baseline examination.

Sensitivity analyses will be based on the per-protocol (PP) population. For the NICC group, the PP population consists in all subjects delivering at least 80% of the planned measurements. For the SoC group, there is no difference between the PP and the FAS population.

Safety will be investigated using the PP population.

### Study population

The electronic trial database was released on 1 December 2017. First patient in was on 4 December 2017. The trial is ongoing. It is expected that recruitment ends by 15 August 2019 with a total number of approximately 950 patients recruited into the trial.

### Screening data

Screening data as required for the Consolidated Standards of Reporting Trials (CONSORT) Figure on the study flow will be provided as summary table by the Universitätsmedizin Rostock Versorgungsstrukturen GmbH.

### Eligibility

Data on eligibility as required by the CONSORT Figure on the study flow will be provided as summary information by the Universitätsmedizin Rostock Versorgungsstrukturen GmbH.

### Recruitment

Information on patients recruited is available from the trial database. All patients who have signed the informed consent form and for whom a record is created in the database are considered recruited. Creation of the record requires successful randomization of the patient.

If patients withdraw their participation, their information is deleted. Such patients are given an entry in the trial database and can be identified as withdrawn. Similarly, if patients need to be excluded from the trial because it turns out that they do not fulfill all inclusion criteria and/or fulfill an exclusion criterion, this is traced in the trial database, and patient data are deleted.

### Withdrawal/follow-up

Patients may withdraw their trial participation at any time without providing reasons for their withdrawal. If the reason for withdrawal is provided, it will be noted.

Follow-up time points are 6 and 12 months after randomization. Patients are considered loss to follow-up if they are not assessed in the study center at 6 and 12 months post randomization or at 12 months post randomization. A time difference of ± 1 calendar month is allowed around a follow-up time point. For example, if a patient has been recruited on 28 February 2018, the window for the 6-month follow-up is from 28 July 2018 to 28 September 2018 (both dates inclusive), and the window for the 1-year follow-up is 28 January 2019 to 28 March 2019 (both dates inclusive).

The information on withdrawal and loss to follow-up will be provided in the CONSORT flow chart.

### Compliance with the investigational plan

Patients in the SoC group adhere to the protocol if they participate in the follow-up visits at 6 and 12 months. A cross-table will be generated for reporting adherence to scheduled assessments by treatment and primary disease.

### Baseline patient characteristics

The type of descriptive statistics used in this trial are described in the following:

- Type M (measurement): median and range with 95% confidence Hodges-Lehmann intervals for the difference of medians
- Type LN (log-normal): type LN statistics are computed for logarithms and converted back to geometric means, ratio of geometric means and coefficients of variation
- Type N (normal): Means and standard deviations (SD) for each treatment group and 95% confidence interval for the difference of means
- Type O (ordinal): absolute and relative frequency distributions and 95% Wald confidence interval for the odds ratio from an ordinal logistic regression on allocated treatment
- Type Po (Poisson): type Po statistics are calculated from a Poisson regression model together with 95% Wald confidence interval allowing for over- or underdispersion
- Type P (proportion): absolute and relative frequencies together with 95% Wilson score confidence intervals for the difference of proportions
- Type T (time to event): Kaplan-Meier curves and hazard ratio (HR) with 95% confidence interval estimated from Cox regression
- Type U (unordered categorical): absolute and relative frequency distributions and 95% Wald confidence interval for the odds ratio from a multinomial logistic regression on allocated treatment

The final SAP provides a complete list of the 54 baseline variables to be reported, and the list starts as follows:

1. Sex: Type P
2. Ethnicity: Type P
3. Migrational background: Type P
4. Marital status: Type U
5. …

### Statistical analysis of primary endpoints

The analyses of the primary endpoints will take into account the stratification variables used in the randomization procedure. Randomization was done in a 1:1 ratio using stratified permuted block randomization (PBR) with variable block length. Stratification variables were diagnosis (AF, HF, TRH) and center (inpatients/outpatients at UMR/Schwerin) [1].

#### First primary endpoint

- *Endpoint*: composite endpoint mortality, stroke or myocardial infarction 1 year after randomization
- *Statistical approach:* logistic regression
- *Adjustment for*: primary disease AF, HF and TRH and center
  Reference category: disease with largest number of randomized patients
  Reference category: largest center
- *Statistical test:* two-sided asymptotic Wald test plus corresponding two-sided 95% confidence intervals

#### Second primary endpoint

- *Endpoint*: number of inpatient days within the 1 year after randomization
- *Statistical approach*: Poisson regression allowing for over- or underdispersion
- *Adjustment for*: primary disease AF, HF and TRH and center
  Reference categories: see previous section
- *Statistical test*: two-sided asymptotic Wald test plus corresponding two-sided 95% confidence intervals

#### Third primary endpoint

- *Endpoint*: composite endpoint mortality, stroke, myocardial infarction or cardiac decompensation 1 year after randomization
- *Statistical approach*: logistic regression
- *Adjustment for*: primary disease AF, HF and TRH and center
  Reference category: see above
- *Statistical test*: two-sided asymptotic Wald test plus corresponding two-sided 95% confidence interval

### Statistical analysis of secondary endpoints

#### Single endpoints from composite endpoints

- *Endpoints*: mortality yes/no, stroke yes/no, myocardial infarction yes/no, cardiac decompensation yes/no, all1 year after randomization
- *Statistical approach*: logistic regression. In case of event rate < 5%: Firth regression
- *Adjustment for*: primary disease AF, HF and TRH and center
  Reference category: disease with largest number of randomized patients
  Reference category: largest center
- *Statistical test*: two-sided asymptotic Wald test plus corresponding two-sided 95% confidence intervals
- *Assumption check*: not necessary

#### Event time endpoints from composite endpoints

- *Endpoints*: time until mortality, stroke, myocardial infarction, cardiac decompensation within 1 year after randomization
- *Statistical approach*: Cox regression
- *Adjustment for*: primary disease AF, HF and TRH and center
  Reference category: disease with largest number of randomized patients
  Reference category: largest center
- *Statistical test*: two-sided asymptotic Wald test plus corresponding two-sided 95% confidence intervals
- *Assumption check*: log rank test

#### Count data endpoints derived from second primary endpoint

- *Endpoints*: number of hospitalizations within 1-year observation period, in months 10–12, in months 4–6, length of hospital stays (days) within 1 year observation period, in months 4–6, in months 10–12, the same for cardiovascular-related reasons
- *Statistical approach*: Poisson regression allowing for over- or underdispersion
- *Adjustment for*: primary disease AF, HF and TRH and center
  Reference category: disease with largest number of randomized patients
  Reference category: largest center
- *Statistical test*: two-sided asymptotic Wald test plus corresponding two-sided 95% confidence intervals
- *Assumption check*: regression analysis with negative binomial model

#### Additional secondary endpoints – quantitative values

- *Endpoints*: EuroQol 5 dimensions, 5 levels health survey (EQ-5D-5 L) index value, HeartQoL, SSUK, Patient Health Questionnaire-9 (PHQ-9), General Anxiety Disorder-7 (GAD-7), World Health Organization-5 (WHO-5), Medication Adherence Rating Scale (MARS-D), Beliefs about preference in dosing Medications Questionnaire (BMQ), all 1 year after randomization. Analyses will be done for index or total scores and domains, except for EQ-5D-5 L, where only the index will be analyzed
- *Statistical approach*: linear regression
- *Adjustment for*: primary disease AF, HF and TRH and center
  Reference category: disease with largest number of randomized patients
  Reference category: largest center
- *Statistical test*: two-sided asymptotic Wald test plus corresponding two-sided 95% confidence intervals
- *Assumption check*: not necessary because of large sample size. Means should be sufficiently close to a normal distribution

#### Additional secondary endpoints – categorical cutoffs

- *Endpoints*: PAM13-D (levels 1–4), PHQ-9 (no depression, mild depression, moderate depression, moderately severe depression, severe depression), EQ-5D-5 L each category, EQ-5D-5 L problems yes/no, GAD-7 (mild, moderate, severe anxiety), WHO-5 score ≤ 50 (poor well-being) all 1 year after randomization
- *Statistical approach*: multinomial or binary logistic regression
- *Adjustment for*: primary disease AF, HF and TRH and center
  Reference category: disease with largest number of randomized patients
  Reference category: largest center
- *Statistical test*: two-sided asymptotic Wald test plus corresponding two-sided 95% confidence intervals
- *Assumption check*: not necessary

#### Physician visits

- *Endpoints*: number of visits to the general physician within 1 year after randomization, number of visits to the cardiologist within 1 year after randomization. Data determined from asking patients
- *Statistical approach*: Poisson regression allowing for over- or underdispersion
- *Adjustment for*: primary disease AF, HF and TRH and center
  Reference category: disease with largest number of randomized patients
  Reference category: largest center
- *Statistical test*: two-sided asymptotic Wald test plus corresponding two-sided 95% confidence intervals
- *Assumption check*: regression with negative binomial model

### Sensitivity analyses

Checks for model assumptions have been described above. Additional sensitivity analyses are not planned.

#### Subgroup analyses – disease-specific secondary endpoints

The three diseases form subgroups, which will be analyzed separately.

#### NYHA stadium – heart failure

- *Endpoints*: NYHA stadium 1 year after randomization
- *Statistical approach*: multinomial logistic regression
- *Adjustment for*: center
  Reference category: largest center
- *Statistical test*: two-sided asymptotic Wald test plus corresponding two-sided 95% confidence intervals
- *Assumption check*: not necessary

#### EHRA stadium – atrial fibrillation

- *Endpoints*: EHRA stadium 1 year after randomization
- *Statistical approach:* multinomial logistic regression
- *Adjustment for:* center
  Reference category: largest center
- *Statistical test*: two-sided asymptotic Wald test plus corresponding two-sided 95% confidence intervals
- *Assumption check*: not necessary

#### CHA_2_DS_2_-VASc score – atrial fibrillation

- *Endpoints*: CHA_2_DS_2_-VASc score 1 year after randomization
- *Statistical approach*: linear regression
- *Adjustment for*: center
  Reference category: largest center
- *Statistical test*: two-sided asymptotic Wald test plus corresponding two-sided 95% confidence intervals
- *Assumption check*: not necessary because of large sample size; means should be sufficiently close to a normal distribution

#### HAS-BLED score – atrial fibrillation

- *Endpoints*: HAS-BLED score 1 year after randomization
- *Statistical approach*: linear regression
- *Adjustment for*: center
  Reference category: largest center
- *Statistical test*: two-sided asymptotic Wald test plus corresponding two-sided 95% confidence intervals
- *Assumption check*: not necessary because of large sample size; means should be sufficiently close to a normal distribution

#### Number of antihypertensive medicines – treatment resistant hypertension

- *Endpoints*: number of antihypertensive medicines being taken 1 year after randomization
- *Statistical approach*: Poisson regression allowing for over- or underdispersion
- *Adjustment for*: center
  Reference category: largest center
- *Statistical test*: two-sided asymptotic Wald test plus corresponding two-sided 95% confidence intervals
- *Assumption check*: regression with negative binomial model

#### Blood pressure – treatment resistant hypertension

- *Endpoints*: blood pressure (in millimeters of mercury (mmHg)) year after randomization
- *Statistical approach*: linear regression
- *Adjustment for*: center
  Reference category: largest center
- *Statistical test*: two-sided asymptotic Wald test plus corresponding two-sided 95% confidence intervals
- *Assumption check*: not necessary because of large sample size; means should be sufficiently close to a normal distribution

#### Additional analyses: exploratory analyses, analyses for publications

Exploratory analyses and analyses for publications will be marked as such.

### Missing data and outliers

#### Original result analysis (CC)

Original result (OR) analysis implies the analysis of data exactly as observed, irrespective whether subjects received the intervention at an observational time point or not. Missing data are not replaced in this analysis. The term complete case (CC) analysis is often used synonymously for this analysis.

#### Multiple imputation (MI)

Primary endpoints will be analyzed using multiple imputation. The following covariates will be used for the multiple imputation of the primary endpoints:

- Sex
- Age at baseline
- Primary disease
- EQ-5D-5 L index at baseline

If all covariates have fewer than 1% missing observations, a simple imputation will be done: for a continuous covariate a random number will be drawn from a normal distribution with mean and standard deviation from the observed distribution. For a dichotomous covariate, a 0 or 1 value will be drawn from the binomial distribution with the success proportion *p* estimated from the observed distribution.

The seed for the imputation is 4242.

If the proportion of missing data exceeds 1% for at least one of the listed variables and if the proportion of missing variables ≤ 20% for all variables, a MI will be done with at least *m* = 10.

The seed for the imputation is 4242.

It is assumed that the data are missing at random (MAR). Both binary and categorical variables will be imputed.

It will be tested whether missing data are missing completely at random (MCAR). To this end, a suitable statistical test will be used to identify a relationship to missingness by treatment allocation and the covariates which will be used for imputation. For continuous covariates, the *t* test will be used, for categorical variables a test based on contingency tables, such as Fisher’s exact test for treatment allocation.

If one of the covariates is significantly associated with missing data, the MCAR assumed is considered to be violated, and missing data are assumed to be MAR. Statistical models assuming missing data to be missing not at random (MNAR) will not be considered. Emphasis is put on the MAR assumption.

#### Last observation carried forward (LOCF)

An alternative imputation approach is to forecast missing data by the last observed value or to forecast the last values by interpolation.

To this end, let *D*_*1*_ be the date of the 12-month visit at which the endpoint *E*_*1*_ is observed. Let *D*_*− 1*_ be the date of the 6-month visit at which the *E*_*−1*_ is observed. Then, the endpoint at time 0 can be interpolated by ${E}_0={E}_{- 1}+\left(\left({E}_1-{E}_{- 1}\right)\times \left({D}_0-{D}_{- 1}\right)/\left({D}_1-{D}_{- 1}\right)\right)$ because endpoints are equally distant.

### Safety data

Missing safety data will not be imputed.

### Safety (harms)

The devices used in this trial are not known to cause adverse device effects. Specifically, devices are used for the following measurements of vital signs: blood pressure, pulse, oxygen saturation or body weight.

Therefore, serious adverse events will be tabulated.

### Blinding

Although originally intended with blinded observer, blinding will not be possible in this trial.

### Software

All analyses will be carried out in R.

### Data cleaning

All data cleaning will be performed by AMEDON GmbH prior to data release. Additional data cleaning is not intended for the electronic case report form (eCRF) data.

### Data merging

Data merging will not be required as all necessary data will be provided as a single file by the data warehouse of AMEDON GmbH.

### Data import and export

Data will be imported from SAS export file, SPSS export file or Excel export file. All data will be stored in the separate directory /R/HerzEffekt/Data.

### Release, transfer and storage of data and program

Final datasets, analysis programs and results will be stored either on CD or on a USB stick in the Trial Master File (TMF).

### Validation of program code

The R code for the primary analysis will be checked by a second scientist. Independent programming by a second person is not intended. Important analysis for publications will also be checked by a second scientist.

### Data management plan

The data management plan is part of the TMF.

### Trial Master File

The TMF is located with the sponsor.

### Statistical Master File

The Statistical Master File (SMF) will be located with the trial biostatistician. It will include the transferred data, the analysis programs and the tables and listings for the analyses described in this SAP.

### Standard operating procedures, recommendations, guidelines and norms

The statistical report will closely follow EN 14155 [2] and the recommendations by Gamble et al. [3].

## Roles in the trial

Coordinating investigator: CS

Deputy coordinating investigator: AÖ

Project manager: KK

Biostatistician: AZ

Sponsor: Universitätsmedizin Rostock Versorgungsstrukturen GmbH, represented by Dipl.-Bwl. Harald Jeguschke, Schillingstr. 35, 18,057 Rostock, Germany.

## Data Availability

The study protocol is published in *Trials* [1]. It is also available upon request from the corresponding authors of the protocol.

## Abbreviations

AF: Atrial fibrillation
CVD: Cardiovascular disease
DMC: Data Monitoring Committee
ESC: European Society of Cardiology
HF: Heart failure
NICC: Novel integrated care concept
RCT: Randomized controlled trial
SoC: Standard of care
TRH: Treatment-resistant hypertension
UMR: University Medical Center Rostock
